# Impact of antibacterials on subsequent resistance and clinical outcomes in adult patients with viral pneumonia: an opportunity for stewardship

**DOI:** 10.1186/s13054-015-1120-5

**Published:** 2015-11-18

**Authors:** Matthew P. Crotty, Shelby Meyers, Nicholas Hampton, Stephanie Bledsoe, David J. Ritchie, Richard S. Buller, Gregory A. Storch, Marin H. Kollef, Scott T. Micek

**Affiliations:** Pharmacy Department, Barnes-Jewish Hospital, One Barnes Jewish Hospital Plaza, St. Louis, MO 63110 USA; St. Louis College of Pharmacy, 4588 Parkview Place, St. Louis, MO 63110 USA; Center for Clinical Excellence, BJC Healthcare, 8300 Eager Road, St. Louis, MO 63144 USA; Department of Pediatrics, Washington University School of Medicine, 4523 Clayton Avenue, Campus Box 8116, St. Louis, MO 63110 USA; Division of Pulmonary and Critical Care Medicine, Washington University School of Medicine, 4523 Clayton Avenue, Campus Box 8052, St. Louis, MO 63110 USA

## Abstract

**Introduction:**

Respiratory viruses are increasingly recognized as significant etiologies of pneumonia among hospitalized patients. Advanced technologies using multiplex molecular assays and polymerase-chain reaction increase the ability to identify viral pathogens and may ultimately impact antibacterial use.

**Method:**

This was a single-center retrospective cohort study to evaluate the impact of antibacterials in viral pneumonia on clinical outcomes and subsequent multidrug-resistant organism (MDRO) infections/colonization. Patients admitted from March 2013 to November 2014 with positive respiratory viral panels (RVP) and radiographic findings of pneumonia were included. Patients transferred from an outside hospital or not still hospitalized 72 hours after the RVP report date were excluded. Patients were categorized based on exposure to systemic antibacterials: less than 3 days representing short-course therapy and 3 to 10 days being long-course therapy.

**Results:**

A total of 174 patients (long-course, n = 67; short-course, n = 28; mixed bacterial-viral infection, n = 79) were included with most being immunocompromised (56.3 %) with active malignancy the primary etiology (69.4 %). Rhinovirus/Enterovirus (23 %), Influenza (19 %), and Parainfluenza (15.5 %) were the viruses most commonly identified. A total of 13 different systemic antibacterials were used as empiric therapy in the 95 patients with pure viral infection for a total of 466 days-of-therapy. Vancomycin (50.7 %), cefepime (40.3 %), azithromycin (40.3 %), meropenem (23.9 %), and linezolid (20.9 %) were most frequently used. In-hospital mortality did not differ between patients with viral pneumonia in the short-course and long-course groups. Subsequent infection/colonization with a MDRO was more frequent in the long-course group compared to the short-course group (53.2 vs 21.1 %; P = 0.027).

**Conclusion:**

This study found that long-course antibacterial use in the setting of viral pneumonia had no impact on clinical outcomes but increased the incidence of subsequent MDRO infection/colonization.

## Introduction

Interactions between viral and bacterial respiratory pathogens have been recognized dating back to the 1918 influenza pandemic [1]. Bacterial pneumonia is a well-recognized serious complication of influenza infections and coinfections are commonplace [2–10]. Respiratory syncytial virus (RSV), parainfluenza viruses, rhinoviruses, and adenoviruses have also been linked to bacterial coinfections in humans [11–18]. Animal studies have suggested synergism between bacterial pathogens and other respiratory viruses [19, 20]. The relationship between viral and bacterial respiratory infections creates a difficult situation for clinicians determining the appropriate use of antimicrobials as they treat hospitalized patients with pneumonia while also trying to minimize the development and selection of resistant organisms.

Respiratory viruses are increasingly recognized as the primary etiology of pneumonia among patients requiring hospitalization (2.7–5 % of pneumonia cases) [21, 22]. Advanced technologies using multiplex molecular assays and PCR improve the diagnostic ability to identify viral pathogens in a timely manner and may impact the use of antibacterials in patients with no bacterial infection identified.

Several studies have investigated the impact of respiratory viral pathogen identification on antibacterial exposure [23–26]. Decreased antibiotic use was observed in two pediatric studies assessing the impact of rapid viral diagnostic tests for respiratory tract infections; however, these results were not mirrored in similar adult studies [23–25]. These studies all used immunofluorescent staining as the primary diagnostic technology. To our knowledge, only one study using PCR-based respiratory virus detection has been reported and found no change in antibacterial use with improved diagnoses for lower respiratory tract infections [26].

Broad-spectrum antibacterial exposure increases the risk of subsequent infections with multidrug-resistant organisms (MDROs) and leads to a vicious cycle of empiric broad-spectrum antibacterials to combat increasingly resistant organisms [27]. We and others have previously shown that patients with culture-negative pneumonia frequently receive treatment with broad-spectrum antibiotics, usually in excess of 5–6 days of therapy despite lack of evidence for a bacterial etiology of infection [28, 29]. It is important to recognize that these studies were performed prior to the availability of rapid viral diagnostics which may have influenced how antibiotics were used during those study periods. Use of new diagnostic technologies for respiratory virus detection could decrease unnecessary antibacterial exposures and subsequent MDRO infections. This study aimed to describe the use of continued empiric antibacterials in patients with known viral pneumonia and to determine the impact of such therapies on subsequent bacterial infections/colonization and clinical outcomes.

## Methods

This was a single-center, retrospective cohort study of patients with a positive respiratory virus panel (RVP) at Barnes-Jewish Hospital (BJH) (a 1300-bed urban academic medical center in St. Louis, MO, USA) between 1 March 2013 and 7 November 2014. The study protocol was approved by the BJH, Washington University, and St. Louis College of Pharmacy Institutional Review Boards.

### Study population

Consecutive patients who were ≥19 years of age and admitted to BJH for ≥24 hours were assessed for study inclusion. Patients were identified through a query of an internal database which tracks respiratory viruses. Patient admissions in which a respiratory virus was identified by FilmArray^®^ respiratory panel (FARP) assay (BioFire Diagnostics, Salt Lake City, UT, USA) were screened for inclusion in this study. Included patients had to meet the study definition of viral pneumonia. Patient admissions were excluded if rhinovirus or enterovirus was identified by nasopharyngeal (NP) swab alone but could also be included if identified from lower respiratory tract specimens. Additionally, patients were excluded if there was a virus identified by RVP within the previous 90 days or if a bacterial pathogen was identified by the respiratory panel. Patients who were transferred from an outside hospital and those who were discharged/died or were made comfort care less than 72 hours after the index RVP report date were also excluded to better evaluate continued empiric antibacterial use in this population.

### Respiratory virus panel

The FARP assay is a multiplexed nucleic acid test capable of simultaneous qualitative detection and identification of multiple respiratory viral and bacterial nucleic acids. The FARP became the primary diagnostic RVP used at BJH in March 2013 for all patients presenting to the hospital with a clinical syndrome consistent with pneumonia, as well as for patients developing a hospital-acquired pneumonia where a viral etiology was a clinical consideration. The FARP is capable of detecting 20 total respiratory pathogens (17 viral and three bacterial): *Bordetella pertussis*, *Chlamydophila pneumoniae*, *Mycoplasma pneumoniae*, adenovirus, coronavirus HKU1, coronavirus NL63, coronavirus 229E, coronavirus OC43, influenza A, influenza A subtype H1, influenza A subtype H3, influenza A subtype 2009 H1, influenza B, human metapneumovirus, parainfluenza 1, parainfluenza 2, parainfluenza 3, parainfluenza 4, RSV, and rhinovirus/enterovirus. The FARP assay cannot reliably differentiate between human rhinovirus and enterovirus due to their genetic similarity. The assay is US Food and Drug Administration-approved for NP swabs, and additional sample types have been validated internally.

### Definitions

Viral pneumonia was defined as identification of a respiratory virus by FARP and a new or progressive radiographic infiltrate within 48 hours of the index RVP. Short-course antibacterial administration was defined as treatment with systemic antibacterials for less than 3 days while long-course therapy was defined as 3–10 days of antibiotics after the index RVP report date with no bacterial infection identified at any point during the admission. MDROs were defined as methicillin-resistant *Staphylococcus aureus* (MRSA), vancomycin-resistant enterococci (VRE), or a bacterium exhibiting in vitro resistance to at least one drug in ≥3 classes of antibacterials for which the organism is not intrinsically resistant [30]. Subsequent isolation of a MDRO was defined as isolation of a study-defined MDRO or a positive epidemiologic screen (MRSA NP swab; VRE stool specimen) ≥10 days after the report date of the index RVP. Positive epidemiologic screens for MRSA or VRE were only considered to be subsequent colonization of an MDRO if the patient had at least one screen in the previous 180 days and all screening tests were negative prior to the index RVP. Immunocompromised status was defined as a diagnosis of human immunodeficiency virus (HIV), active malignancy (stem cell transplant or receiving chemotherapy), solid organ transplant, or currently on immunosuppressive therapy (prednisone 20 mg/day for at least 30 days or equivalent).

### Microbiologic evaluation

Information regarding the FARP including time of collection/report, type of specimen, patient location at time of collection, and resulting findings were obtained from an internal database. Additionally, all available aerobic and anaerobic bacterial cultures were evaluated based on electronic medical record (EMR) query. In vitro susceptibilities of isolated bacterial pathogens were evaluated as reported per institutional practices. Urine *Legionella* antigen, direct-fluorescent antibody for *Pneumocystis jiroveci*, and *Clostridium difficile* toxin assay were also evaluated.

### Patient characteristics and antibacterial exposure

Data collected to describe patient groups included demographic information, comorbid conditions, and clinical outcomes. Charlson’s comorbidity index was used as a summative score of underlying disease states [31]. Antibacterial exposures were calculated using EMR orders for systemic antibacterials. Days of therapy (DOT) and DOT normalized per 1000 patient-days (DOT/1000PD) were calculated as described previously [32]. EMR queries were used to acquire patient information where possible. Manual chart review was used to validate and supplement all EMR queries.

### Endpoints

The primary endpoint was subsequent isolation of a MDRO ≥10 days after the index RVP report date. In-hospital mortality and readmission at 30, 90, and 180 days were secondary endpoints.

### Statistical analysis

Dichotomous variables were compared using the chi-square test or Fisher’s exact test as appropriate. Continuous variables were compared using the Mann–Whitney *U* test. All tests were two-tailed and *P* <0.05 was considered significant. Univariate analyses were performed to compare the group that received long-course antibacterials and the group that received short-course antibacterial therapy. Kaplan–Meier survival analysis was used to compare risk of in-hospital mortality between comparator groups while censoring for patient discharge. All statistical analyses were performed using SPSS software (IBM SPSS Statistics, version 22.0; Chicago, IL, USA).

## Results

### Cohort description

**Fig. 1:**
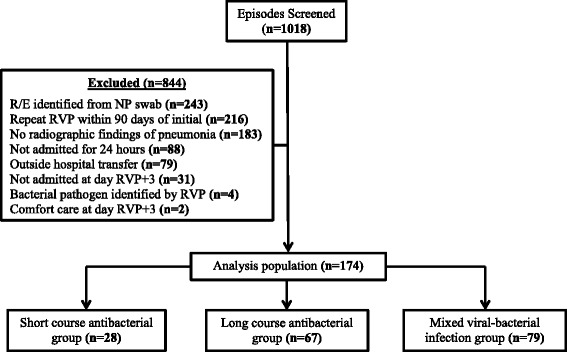
Identification of study population. Short-course antibacterial group did not receive empiric antibacterials from RVP+3 to RVP+10, long-course antibacterial group received empiric antibacterials between RVP+3 and RVP+10, and mixed viral–bacterial infection group included patients with both identification of a respiratory virus and a bacterial pathogen. *NP* nasopharyngeal, *R/E* rhinovirus/enterovirus, *RVP* respiratory virus panel, *RVP+3* 3 days after index RVP, *RVP+10* 10 days after index RVP

**Fig. 2:**
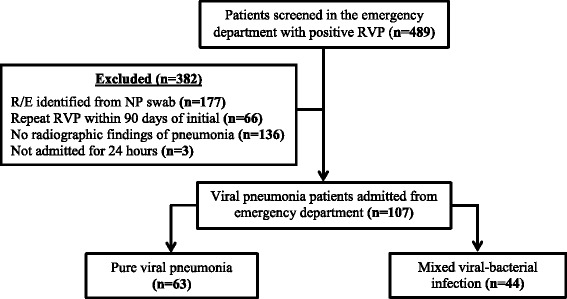
Patients with pure viral pneumonia or mixed viral–bacterial infection as screened in the emergency department. Pure viral pneumonia group included patients with identification of a respiratory virus only. Mixed viral–bacterial infection group included patients with both identification of a respiratory virus and bacterial pathogen. *NP* nasopharyngeal, *R/E* rhinovirus/enterovirus, *RVP* respiratory virus panel

A total of 174 consecutive patients (long course, *n* = 67; short course, *n* = 28; mixed bacterial–viral infection, *n* = 79) were included in this study (Fig. 1). Demographic and clinical characteristics are summarized in Table 1. The median age was 57 years and 46.0 % were male. A majority of patients in the cohort were deemed to be immunocompromised (56.3 %), with active malignancy the most common etiology (69.4 %). Only 5 of 174 patients had been admitted within the 90 days prior to the index admission. Figure 2 provides a breakdown of the subgroup of patients screened in the emergency department setting according to the presence of pure viral and mixed infections.

**Table 1:** Demographic and clinical characteristics of patients with viral pneumonia according to empiric antibacterial therapy

	Long course	Short course	Mixed infection
	(*n* = 67)	(*n* = 28)	(*n* = 79)
Age	55 (45–66)	57.5 (45–65)	56 (45–69)
Male	33 (49.3)	15 (53.6)	32 (40.5)
Caucasian	43 (64.2)	17 (60.7)	45 (57.0)
African American	20 (29.9)	7 (25)	30 (38.0)
Weight (kg)	81.3 (65.5–95.3)	74.3 (67.5–104.2)	73.5 (55.1–88.7)*
BMI	27.6 (22.6–35.1)	27.3 (22.7–33.8)	25.8 (20.1–32.0)*
Prior admit within 90 days	2 (3.0)	0 (0)	3 (3.8)
Chronic heart failure	12 (17.9)	6 (21.4)	12 (15.2)
Coronary artery disease	13 (19.4)	4 (14.3)	15 (19.0)
COPD	12 (17.9)	9 (32.1)	16 (20.3)
Diabetes mellitus	25 (37.3)	8 (28.6)	25 (31.6)
Chronic kidney disease	15 (22.4)	5 (17.9)	21 (26.6)
ESRD on hemodialysis	6 (9.0)	0 (0)	7 (8.9)
Cirrhosis	2 (3.0)	0 (0)	1 (1.3)
Human immunodeficiency virus	1 (1.5)	0 (0)	2 (2.5)
Active malignancy	31 (46.3)	11 (39.3)	26 (33.0)
Stem cell transplant	14 (20.9)	5 (17.9)	10 (12.7)
Solid organ transplant	9 (13.4)	2 (7.1)	9 (11.4)
Lung	4 (6.0)	2 (7.1)	8 (10.1)
Heart	2 (3.0)	0 (0)	1 (1.3)
Kidney	3 (4.5)	0 (0)	0 (0)
Immunosuppressive therapy	1 (1.5)	3 (10.7)	8 (10.1)*
Immunocompromised	39 (58.2)	15 (53.6)	44 (55.7)
CCI	4 (3–6)	3 (1–5)	4 (2–6)
Intensive care unit admit	44 (65.7)	17 (60.7)	63 (79.7)
Oncology^a^	18 (26.9)	5 (17.9)	16 (20.3)
Nononcology	26 (38.8)	12 (42.9)	47 (59.5)*
Mechanical ventilation	34 (50.7)	10 (35.7)	53 (67.1)**
Days	5.9 (3.4–13.7)	3.7 (1.85–27.2)	7.0 (3.3–11.9)*
Central line placed	56 (83.6)	14 (50)	53 (67.1)*
Vasopressor support	14 (20.9)	4 (14.3)	35 (44.3)*^,^**
Days	3.55 (1.93–13.1)	2.2 (0.8–10.6)	6.3 (3.4–11.6)
APACHE II score	15 (11–20)	14 (8–17)	19 (14–23)*^,^**

Data expressed as number (% of total) or median (interquartile range)

^a^ICU admit was determined to be oncology related if the patient fit the study definition for active malignancy

*Statistically significant difference (*P* <0.05) between long-course and mixed-infection groups

**Statistically significant difference (*P* <0.05) between short-course and mixed-infection groups

*BMI* body mass index, *CCI* Charlson’s comorbidity index, *COPD* chronic obstructive pulmonary disease, *ESRD* end-stage renal disease

Rhinovirus/enterovirus (23.0 %), influenza (19.0 %), and parainfluenza (15.5 %) were the viruses most commonly identified in the cohort (Table 2). Of the total 174 patients, 11 had multiple respiratory viruses identified by RVP: RSV (5 of 11), rhinovirus/enterovirus (5 of 11), influenza (4 of 11), human metapneumovirus (3 of 11), parainfluenza (2 of 11), coronavirus (2 of 11), and adenovirus (2 of 11). Specimens resulting positive for a respiratory virus in the cohort included NP swabs (40.8 %), bronchoalveolar lavage (25.9 %), tracheal aspirates (18.4 %), bronchial washes (9.8 %), and sputum samples (4.0 %). The RVP identified a respiratory virus from multiple specimens in 25 patients. Among the 25 patients with multiple positive specimens, the most common were bronchoalveolar lavage (19 of 25 patients) and tracheal aspirates (13 of 25 patients).

**Table 2:** Viruses identified by FA RP in patients with viral pneumonia according to empiric antibacterial therapy

	Long course	Short course	Mixed infection
	(*n* = 67)	(*n* = 28)	(*n* = 79)
Rhinovirus/enterovirus	15 (22.4)	6 (21.4)	19 (24.1)
Influenza	14 (20.9)	4 (14.3)	15 (19.0)
Parainfluenza	12 (17.9)	7 (25)	8 (10.1)
Respiratory syncytial virus	8 (11.9)	3 (10.7)	8 (10.1)
Human metapneumovirus	8 (11.9)	3 (10.7)	6 (7.6)
Coronavirus	6 (9.0)	2 (7.1)	8 (10.1)
Adenovirus	3 (4.5)	1 (3.6)	7 (8.9)
Multiple viruses	1 (1.5)	2 (7.1)	8 (10.1)

Data expressed as number (% of total)

No statistically significant differences were found between groups

### Patient group characteristics

No significant differences in comorbidities between groups were identified (Table 1). Patients with mixed viral–bacterial infection had statistically greater APACHE II scores and were more likely to require vasopressor support compared with patients with pure viral infection. There was no significant difference in the number of immunocompromised patients between groups, although numerically more patients who received long-course antibacterials had an active malignancy or solid organ transplant compared with patients receiving short-course therapy. Virus types identified by FARP assay were similar in all three patient groups (Table 2). The bacterial coinfecting organisms identified in patients with mixed viral–bacterial infection are presented in Table 3. Respiratory coinfection with a bacterial pathogen was most common, with *S. aureus*, *Streptococcus pneumoniae*, and *Pseudomonas aeruginosa* accounting for the most frequent respiratory coinfecting bacteria.

**Table 3:** Coinfecting bacterial organisms identified in patients with viral pneumonia

	Mixed infection
	(*n* = 79)
Corespiratory infection	52 (65.8)
*Staphylococcus aureus*	25 (31.6)
MSSA	12 (15.2)
MRSA	19 (24.1)
Streptococci	10 (12.7)
*Streptococcus pneumoniae*	9 (11.4)
*Pseudomonas aeruginosa*	8 (10.1)
Enterobacteriaceae	5 (6.3)
Urinary tract infection	17 (21.5)
Enterococci	8 (10.1)
Enterobacteriaceae	8 (10.1)
*P. aeruginosa*	1 (1.3)
Skin and skin structure infection	8 (10.1)
Intra-abdominal infection	2 (2.5)

Data expressed as number (% of total)

*MRSA* methicillin-resistant *Staphylococcus aureus*, *MSSA* methicillin-susceptible *Staphylococcus aureus*

### Antibacterial use

**Fig. 3:**
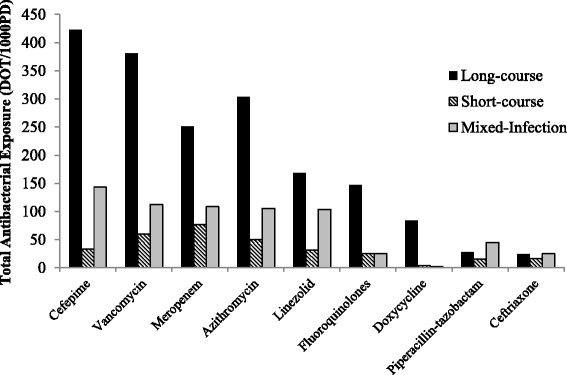
Total antibacterial use among patients with viral pneumonia according to empiric antibacterial therapy groups and mixed infection. *DOT/1000PD* days of therapy normalized per 1000 patient-days

A total of 13 different systemic antibacterials were used as empiric treatment in patients with viral pneumonia without bacterial coinfection for a total of 466 DOT. Vancomycin (50.7 %), cefepime (40.3 %), azithromycin (40.3 %), meropenem (23.9 %), and linezolid (20.9 %) were the most frequently used empiric antibacterials in patients with viral pneumonia without bacterial coinfection (Fig. 3). The most common regimens used in viral pneumonia without bacterial coinfection were vancomycin plus cefepime (28.4 %) and vancomycin plus meropenem (13.4 %). A total of 44 (65.7 %) patients with viral pneumonia without bacterial coinfection received empiric MRSA coverage with vancomycin or linezolid. Empiric antibacterial therapy was continued for a median of 4.1 days (interquartile range, 2.5–6.1 days) in viral pneumonia without bacterial coinfection, with most (69 %) being days on intravenous antibacterials.

Total antibacterial exposure differed between the long-course and short-course groups at 2116 and 484 DOT/1000PD, respectively (Fig. 3). Patients with mixed viral and bacterial infections received a total of 780 DOT/1000PD of systemic antibacterials. Median total antibacterial DOT/1000PD was also significantly higher in the long-course group compared with the short-course group (12.2 vs. 6.4; *P* <0.001) and the mixed-infection group (12.2 vs. 6.3; *P* <0.001). The most common antibacterials used were similar between groups: cefepime (long-course group: 73.1 %; short-course group: 50 %; mixed-infection group: 58.2 %), meropenem (long-course group: 37.3 %; short-course group: 32.1 %; mixed-infection group: 43.0 %), and linezolid (long-course group; 31.3 %; short-course group: 25 %; mixed-infection group: 41.7 %). Vancomycin was more commonly used in the long-course group compared with the mixed-infection group (80.6 vs. 59.5 %; *P* = 0.007) but not compared with the short-course group (80.6 vs. 57.1 %; *P* = 0.081). Azithromycin use was less prevalent in the mixed-infection group compared with the long-course group (48.1 % vs. 67.2 % of patients; *P* = 0.029) and the short-course group (48.1 vs. 71.4 %; *P* = 0.047).

### Clinical outcomes

The number of patients with subsequent MDRO colonization or infection was not significantly different between groups (Table 4). However, in instances of subsequent infection or colonization, where a single patient could have more than one organism, there was a higher rate of MDRO identification among isolates from the group that received long-course antibacterials compared with the group receiving short-course therapy (53.2 vs. 21.1 %; *P* = 0.027) (Table 4). VRE (35 %), coagulase-negative *Staphylococcus* sp. (15 %), *Escherichia coli* (10 %), *Enterobacter cloacae* (10 %), and *Stenotrophomonas maltophilia* (10 %) were the most commonly isolated MDROs in the long-course group. Other MDROs isolated from this group included *Klebsiella pneumonia*e, *Serratia marcescens*, *S. pneumoniae*, and *Achromobacter* sp. The three subsequent infecting MDROs in the short-course group were VRE, *E. cloacae*, and *Corynebacterium* sp. One patient in the long-course group and two patients in the mixed-infection group developed *C. difficile* infection 1 week after empiric antibacterial exposure.

**Table 4:** Clinical outcomes of patients with viral pneumonia according to empiric antibacterial therapy

	Long course	Short course	Mixed infection
	(*n* = 67)	(*n* = 28)	(*n* = 79)
Instances of subsequent MDRO infection or colonization	25/47 (53.2)	4/19 (21.1)*	20/53 (37.7)
MRSA colonization	2/10 (20)	0/7 (0)	3/15 (20)
VRE colonization	3/6 (50)	1/4 (25)	9/20 (45)
MDRO infection	20/31 (64.5)	3/8 (37.5)	8/18 (44.4)
Patients with subsequent MDRO infection or colonization	16 (23.9)	3 (10.7)	16 (20.3)
MRSA colonization	2 (3.0)	0 (0)	3 (3.8)
VRE colonization	3 (4.5)	1 (3.6)	9 (11.4)
MDRO infection	13 (19.4)	3 (10.7)	7 (8.9)
Subsequent *Clostridium difficile* infection	1 (1.5)	0 (0)	2 (2.5)
In-hospital mortality	8 (11.9)	3 (10.7)	23 (29.1)**
Non-ICU	1/23 (4.3)	0/11 (0)	0/16 (0)
ICU (all)	7/44 (15.9)	3/17 (17.6)	23/63 (36.5)**
ICU – oncology	4/18 (22.2)	2/5 (40)	10/16 (62.5)**
ICU – nononcology	3/26 (11.5)	1/12 (8.3)	13/47 (27.7)
Hospital LOS (days)	11 (7–25)	8.5 (5–20)	17 (8.5-28) ***
ICU LOS (days)	8.1 (4.9-17.9)	4.2 (2.8-14.7)*	12 (5.1-19.1) ***
Readmit within			
30 days	14 (20.9)	4 (12.9)	19 (24.0)
90 days	22 (32.8)	8 (25.8)	27 (48.2)
180 days	30 (44.8)	11 (35.5)	29 (51.8)

Data expressed as number (% of total) or median (interquartile range)

*LOS* length of stay, *MDRO* multidrug-resistant organism, *MRSA* methicillin-resistant *Staphylococcus aureus*, *VRE* vancomycin-resistant enterococci

*Statistically significant difference (*P* <0.05) between short-course and long-course groups

**Statistically significant difference (*P* <0.05) between long-course and mixed-infection groups

***Statistically significant difference (*P* <0.05) between short-course and mixed-infection groups

**Fig. 4:**
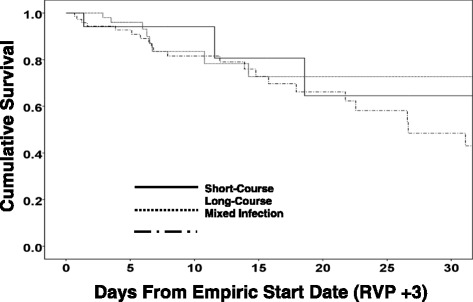
Kaplan–Meier survival analysis among patients with viral pneumonia according to antibacterial treatment course and mixed viral–bacterial infection. Log-rank test: short course versus long course, *P* = 0.854; short course versus mixed infection, *P* = 0.349; long course versus mixed infection, *P* = 0.152. *RVP +3* = 3 days after index respiratory viral panel

In-hospital mortality was statistically higher for the mixed-infection group compared with the long-course therapy group (Table 4). Kaplan–Meier survival analysis showed that the mixed-infection group had the lowest overall survival, but these differences were not statistically significant (Fig. 4). ICU mortality was also significantly higher for patients in the mixed-infection group compared with the long-course therapy group. Patients receiving long-course therapy or those with mixed infection had statistically longer ICU length of stay compared with patients receiving short-course therapy. Hospital readmission rates were similar between groups at 30, 90, and 180 days after index hospitalization discharge.

## Discussion

This study compared a cohort of 174 patients with viral pneumonia and mixed viral–bacterial infection based on exposure to continued empiric antibacterials after respiratory virus identification. More of the subsequent infecting or colonizing bacterial isolates from the group with pure viral pneumonia who received continued long-course antibacterials were defined as MDROs compared with the short-course group (*P* = 0.027). These findings suggest that more prolonged exposure to broad-spectrum antibacterials in patients with viral pneumonia may have promoted resistance in these patients.

No benefit of continued empiric antibacterials for patients with pure viral pneumonia was seen in this study. The risk of bacterial coinfection in the setting of viral pneumonia, especially with influenza, creates a challenging situation for clinicians. The potential detrimental impact of not treating a bacterial pathogen weighs heavily on the decision process and downstream effects of such therapies may be disregarded. Our findings of similar clinical outcomes between patients with pure viral pneumonia who received long-course antibacterials after virus recognition and those who did not may suggest opportunity for de-escalation of empiric antibacterial therapy when viral pneumonia is identified.

A previous randomized controlled trial by Oosterheert et al. [26] evaluated implementation of real-time PCR rapid diagnostics for respiratory pathogen identification. They found increased diagnostic yield with the assay but no difference in antibiotic use, and hypothesized that reluctance to change treatment based on testing results may have inhibited cost-effectiveness from being demonstrated. In our study, systemic antibacterials were discontinued following identification of a respiratory virus by RVP for several patients; however, whether virus identification directly led to discontinuation of antibacterials cannot be determined. The willingness of prescribers to de-escalate and stop antibacterials in this setting may suggest increased recognition of the role of viral pathogens in pneumonia. Additionally, the expanded panel of viruses detected may have factored into how results were perceived, as prescribers may have been more likely to attribute pneumonia to newly detectable viruses such as human metapneumovirus. However, it is not possible to definitively determine the rationale for stopping antibacterial therapy.

Timely antibiotic administration is crucial for treating hospitalized patients with suspected pneumonia [33]. Antimicrobial de-escalation attempts to balance the use of these essential drugs up front with the emergence of resistance [34]. The optimal strategy for de-escalation of antibacterials in the setting of viral pneumonia without an identified bacterial coinfection is unclear. Our study found no difference in clinical outcomes based on antibiotic duration of therapy in patients with viral pneumonia despite significantly different total antibacterial exposure (DOT/1000PD) between groups. Byington et al. [24] found previously that improved diagnostic technologies enhancing detection of respiratory viruses decreased antibacterial use at a children’s hospital. The authors concluded that improved diagnostics are an important tool in decreasing unnecessary antibacterial prescribing. Our study similarly illustrated the potential impact of respiratory virus diagnostics on antibacterial use in an adult population.

*C. difficile* infection is a major cause of morbidity and mortality in US hospitals and has been directly linked to exposure to broad-spectrum antibiotics [35, 36]. In a cohort of hospitalized adult patients, Shiley et al. [37] found that significantly more patients who continued to receive antibacterials after diagnosis of a viral respiratory tract infection developed *C. difficile* infection. One patient in our study who was treated with long-course antibacterials after identification of a respiratory virus also developed *C. difficile* infection. Strategies to best limit the use of unneeded antibacterials are important to curtail against the growing issues of *C. difficile* and resistance, and may be aided by de-escalation approaches using enhanced viral diagnostic technologies.

Limitations of this study should be noted. First, this was a small retrospective cohort study of patients at a single institution and may not be representative of all settings. It is important to note that BJH is a regional specialty referral hospital and not a community hospital. This accounts for the case mix with a high prevalence of immunosuppressed patients and the low prevalence of narrow spectrum empiric antibiotic utilization. The small number of patients meeting inclusion criteria did not allow for definitive conclusions to be made regarding group comparison as a lack of statistically significant differences being found could be due to the lack of sample size. Second, patients were determined to have viral pneumonia based on virus identification and radiographic findings but other markers of illness, such as white blood cell count and fever, were not considered and the retrospective nature of the study did not allow evaluation of what drove continuation of antibacterials in some patients but not others. Moreover, we did not attempt to identify risk factors associated with pure viral pneumonia. Third, although coinfecting bacterial pathogens were not identified in patients with pure viral pneumonia, it is impossible to prove that they were not present. Receipt of antibacterials prior to obtaining bacterial cultures could have limited the diagnostic yield of bacterial cultures in some cases and yield from bacterial cultures is not perfect. Finally, all of the viral pneumonia cases occurred in a 20-month period. Viral epidemiology during this time may not be representative of all seasons. Influenza H1N1 p2009 was the primary influenza virus identified in our study (85 %). Incidence rates of bacterial coinfection and coinfecting organisms may differ from year to year and from virus type to virus type, which may hinder application of de-escalation strategies using the results of this study.

It is not possible to directly link the development of subsequent MDRO infections/colonization and *C. difficile* infection seen in our study to the continued empiric antibacterials administered. All of the patients included in the cohort received antibacterials at some point during their index hospitalization and infection control measures were not directly assessed in these patients. Additionally, hospitalization itself probably increases the risk of these patients being colonized with MDROs. Use of cephalosporins and vancomycin, two of the most commonly administered empiric agents in our study, have been implicated as increasing the prevalence of VRE, the most commonly identified subsequent MDRO in this study [38, 39]. Decreasing exposure to broad-spectrum antibacterials such as third-generation and fourth-generation cephalosporins and vancomycin would be expected to lessen the incidence of VRE and other MDROs as was seen in this study, but the risk of development and transmission of resistance in the hospital cannot be completely eliminated. Antibacterials are extraordinarily important in the treatment of many hospitalized patients and their use is often warranted. Decreasing unnecessary use may help curb acquirement of resistant organism in healthcare settings but even appropriate use can lead to the development of resistance. Only through multifaceted efforts of infection control and antimicrobial stewardship can the spread of MDROs between patients, clinicians, workers, and visitors be diminished.

## Conclusions

This study highlights the potential benefits of improved diagnostics for respiratory viruses, primarily the potential for decreased antibacterial exposure and thus decreased selective pressure for resistant bacterial isolates. Antibacterial exposure applies selective pressure and promotes colonization/infection by resistant organisms including MRSA and VRE [40, 41]. Halting this process is essential to maintain effective therapeutic options in the future and may be aided by discontinuation of antibacterials in cases of viral pneumonia. In our study, patients with viral pneumonia exposed to long-course antibacterials had more occurrences of subsequent infection or colonization with MDRO isolates. In contrast, the number of patients with subsequent MDRO infection or colonization was not different between groups although this may be due to the small number of patients in each group. No differences in clinical outcomes, including in-hospital mortality and readmission rates, were observed between patient groups. In the setting of viral pneumonia and no coinfecting bacterial pathogens, discontinuation of antibacterials is reasonable in many if not most cases, and may allow for decreased overall antibacterial use. Enhanced diagnostic technologies can potentially be incorporated into antimicrobial stewardship practices to allow for de-escalation of unnecessary antibacterials. These findings warrant further investigation to determine the applicability of an antibacterial de-escalation approach in the setting of viral pneumonia.

## Key messages

- In this single-center retrospective cohort, patients with viral pneumonia who continued to be treated with systemic antibacterials 3 days after virus identification were more likely to have a subsequent infection or colonization with a MDRO than were patients in whom systemic antibacterials were stopped.
- In-hospital mortality based on Kaplan–Meier survival analysis and readmission rates were not different between groups based on antibacterial exposure after respiratory virus identification.

## Abbreviations

BJH: Barnes-Jewish Hospital
DOT: Days of therapy
DOT/1000PD: Days of therapy normalized per 1000 patient-days
EMR: Electronic medical record
FARP: FilmArray^®^ respiratory panel
HIV: Human immunodeficiency virus
MDRO: Multidrug-resistant organism
MRSA: Methicillin-resistant *Staphylococcus aureus*
NP: Nasopharyngeal
RSV: Respiratory syncytial virus
RVP: Respiratory virus panel
VRE: Vancomycin-resistant enterococci
